# Beyond drug discovery: Exploring the physiological and methodological dimensions of zebrafish in diabetes research

**DOI:** 10.1113/EP091587

**Published:** 2024-01-27

**Authors:** Tamsheel Fatima Roohi, Syed Faizan, Mohd. Farooq Shaikh, Kamsagara Linganna Krishna, Seema Mehdi, Nabeel Kinattingal, Alina Arulsamy

**Affiliations:** ^1^ Department of Pharmacology JSS College of Pharmacy JSS Academy of Higher Education and Research Mysuru Karnataka India; ^2^ Department of Pharmaceutical Chemistry JSS College of Pharmacy JSS Academy of Higher Education and Research Mysuru Karnataka India; ^3^ School of Dentistry and Medical Sciences Charles Sturt University Orange New South Wales Australia; ^4^ Neuropharmacology Research Laboratory Jeffrey Cheah School of Medicine and Health Sciences Monash University Malaysia Bandar Sunway Selangor Malaysia

**Keywords:** animal model, anti‐diabetic drugs, *Danio rerio*, diabetes mellitus, hyperglycaemic pathologies

## Abstract

Diabetes mellitus is a chronic disease that is now considered a global epidemic. Chronic diabetes conditions include type 1 and type 2 diabetes, both of which are normally irreversible. As a result of long‐term uncontrolled high levels of glucose, diabetes can progress to hyperglycaemic pathologies, such as cardiovascular diseases, retinopathy, nephropathy and neuropathy, among many other complications. The complete mechanism underlying diabetes remains unclear due to its complexity. In this scenario, zebrafish (*Danio rerio*) have arisen as a versatile and promising animal model due to their good reproducibility, simplicity, and time‐ and cost‐effectiveness. The Zebrafish model allows us to make progress in the investigation and comprehension of the root cause of diabetes, which in turn would aid in the development of pharmacological and surgical approaches for its management. The current review provides valuable reference information on zebrafish models, from the first zebrafish diabetes models using genetic, disease induction and chemical approaches, to the newest ones that further allow for drug screening and testing. This review aims to update our knowledge related to diabetes mellitus by gathering the most authoritative studies on zebrafish as a chemical, dietary and insulin induction, and genetic model for diabetes research.

## Abstract (highlights)

- **What is the topic of this review?**
  Zebrafish as a versatile and superior animal model for mechanistic and therapeutic studies of diabetes and hyperglycaemic pathologies.
- **What advances does it highlight?**
  Zebrafish offer numerous advantages, including a sequenced genome, ease of genetic manipulation, high fecundity, external fertilization, short life cycle and transparent embryos. Zebrafish genetic‐, chemical‐ and diet‐induced hyperglycaemic models have made it possible to advance the understanding of diabetes, diabetic pathology mechanisms and anti‐diabetic drug screening.

## 1 INTRODUCTION

Diabetes mellitus (DM) is considered a global epidemic. According to the World Health Organization, the number of people with diabetes rose from 108 million in 1980 to 422 million in 2014, and there is a globally agreed target to halt the rise in diabetes and obesity by 2025. The majority of people suffering from diabetes live in low‐ and middle‐income countries and 1.5 million deaths are directly attributed to diabetes each year (Reddy, 2022). Type 1 diabetes mellitus (T1DM) results from autoimmune β‐cell destruction in the pancreas, which leads to a complete lack of insulin production (Chen & Liu, 2022). In type 2 diabetes mellitus (T2DM), cells in the body become resistant to the action of insulin, and the pancreas is unable to make enough insulin to overcome this resistance (Deshpande et al., 2008). As a result of long‐term uncontrolled high levels of glucose, diabetes can progress to hyperglycaemic pathologies, including macrovascular and microvascular diseases. Examples of diabetic macrovascular pathologies include cardiovascular diseases, such as coronary artery disease, stroke, arrhythmias and peripheral arterial disease (Aronson, 2008), and diabetic microvascular pathologies include retinopathy, nephropathy and neuropathy. Retinopathy affects the eyes and can lead to visual loss or blindness (Engerman, 1989), nephropathy is a type of kidney disease that can lead to kidney damage and failure (Fineberg et al., 2013), and neuropathy constitutes a group of clinical syndromes caused by damage to the peripheral and autonomic nervous systems that can cause numbness, tingling and other sensory disturbances (Feldman et al., 2019). Other diabetic complications include diabetic ketoacidosis, characterized by high levels of ketones due to a lack of insulin (French et al., 2019), and hyperosmolar hyperglycaemic syndrome, a severe complication characterized by extremely high blood glucose levels, high serum osmolality and dehydration (Pasquel & Umpierrez, 2014). Proper management and control of blood glucose levels through lifestyle modifications, medication and insulin therapy can help prevent or delay the onset of diabetic complications.

**FIGURE 1:**
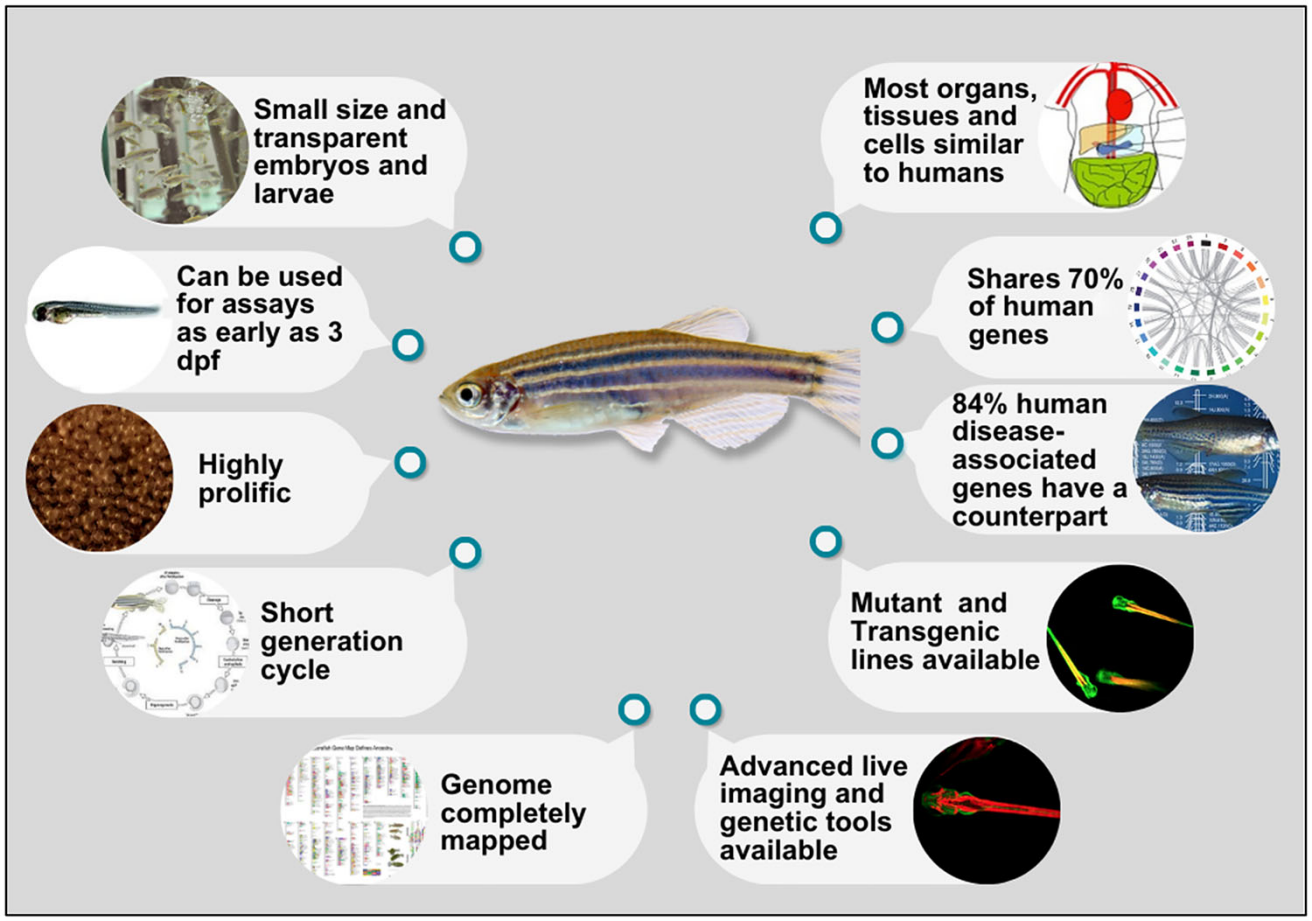
Zebrafish have unique features that make them an ideal biomedical research model. They are a superior animal model for understanding vertebrate embryogenesis, studying human developmental and metabolic diseases, evaluating drug toxicity and predicting drug effects in mammals. Particularly, the similarity of the zebrafish pancreas to the human pancreas in structure and function makes them an ideal model for diabetes mellitus and hyperglycaemic pathology research.

Due to the complexity of diabetes pathology, the complete mechanism remains unclear. Further investigation is required to comprehend the root cause of diabetes, which would aid in the development of pharmacological and surgical approaches for its management. In this scenario, animal models for diabetes research have been used for decades (King, 2012), including mice, rats (Robinson et al., 2019) and fish (Ishikawa, 2000). However, among animal models, zebrafish (*Danio rerio*) has gained popularity and risen as a versatile and promising model (Ribas & Piferrer, 2014). Zebrafish offer unique features, such as less cumbersome housing, external fertilization with high egg production every 7 days, early development allowing the use of embryos and larvae for experimentation as early as 3 days post‐fertilization (dpf) (Kimmel et al., 2015), and a relatively short generation cycle of about 3 months until becoming adults (Goldsmith & Jobin, 2012). The small size and transparent nature of embryos and larvae along with screening and imaging techniques make zebrafish highly convenient for observing rapid external development. Zebrafish and humans share high genetic and physiological homology, and nearly 70% of human diabetes‐related genes have zebrafish orthologues (https://www.sanger.ac.uk/data/zebrafish‐genome‐project/) (Howe et al., 2013). The possibility of knocking in or knocking out genes of interest leads to the obtention of mutant zebrafish lines that serve as biomedical models to study human diseases, which together with modern genome editing tools bring up promising technologies that may hasten the transgenic process. In addition, the zebrafish research community benefits from an up‐to‐date database of techniques, genetic strains and other useful resources (available at http://zfin.org/). The advancement in live imaging along with automated systems and some other beneficial characteristics (Figure 1) have also contributed to making the zebrafish a top choice model for drug screening and toxicity testing. For instance, their small eggs can be placed in the wells of plates and survive for approximately 7 days without feeding or changing the water, facilitating the screening of drugs in a short period and reducing the number of tested drugs (Kimura et al., 2014).

Despite these benefits, assessing substance solubility is a barrier for some zebrafish embryo research, where the tested drugs must be water‐soluble or at least soluble in dimethyl sulfoxide or acetone. When utilizing adult zebrafish, however, this will not be a constraint because force‐feeding can be applied, allowing for the evaluation of the complete sample. Aside from that, the volume of zebrafish blood is fairly limited; however, this limitation can be overcome by applying high‐end analytical methods for metabolite detection, such as liquid chromatography–tandem mass‐spectrometry. The small size of zebrafish may be another disadvantage when performing ablation because it requires more work and skill to extract the organs than in typical mammalian models, though it is possible as demonstrated in a pioneer study for the ablation of β‐cells from the pancreas of zebrafish. In general, the most difficult aspect of establishing diabetes in zebrafish is the rapid recovery of β‐cells. To overcome this limitation, some studies have used chemical and physical ablation of β‐cells in a transgenic genotype with varying results, opening the possibility of crossing this transgenic line with other lines to specifically study almost any zebrafish organ or system (Moss et al., 2009; Yin et al., 2015).

Aside from these limitations in researching diabetic metabolic dysfunction, in addition to the various benefits listed above, zebrafish offer a highly relevant model due to their notable similarities to humans in pancreatic anatomy and function. Human and zebrafish pancreases are morphologically comparable; both contain exocrine and endocrine compartments with similar kinds of cells (Tehrani & Lin, 2011). Likewise, the signalling pathways and mechanisms of zebrafish endocrine pancreas are highly homologous to those of mammals. In addition, the development and function of other tissues, organs and systems involved in glucose homeostasis, such as the brain, liver, adipocyte tissue and skeletal muscle, are also conserved between zebrafish and mammals. The conservation of the pancreatic structure and glucose homeostasis system makes zebrafish useful for identifying novel targets in pancreas‐related diseases such as diabetes (Seth et al., 2013). Therefore, this article provides an authoritative and comprehensive review of the main studies conducted using diabetic zebrafish models based on chemical, dietary and insulin induction, genetic, and surgical approaches over the last 16 years, to identify updated knowledge in diabetes mellitus and its complications, and identify work that will facilitate screening of drugs targeted to such metabolic diseases.

## 2 ZEBRAFISH MODELS FOR DIABETES AND DIABETIC PATHOLOGIES

**FIGURE 2:**
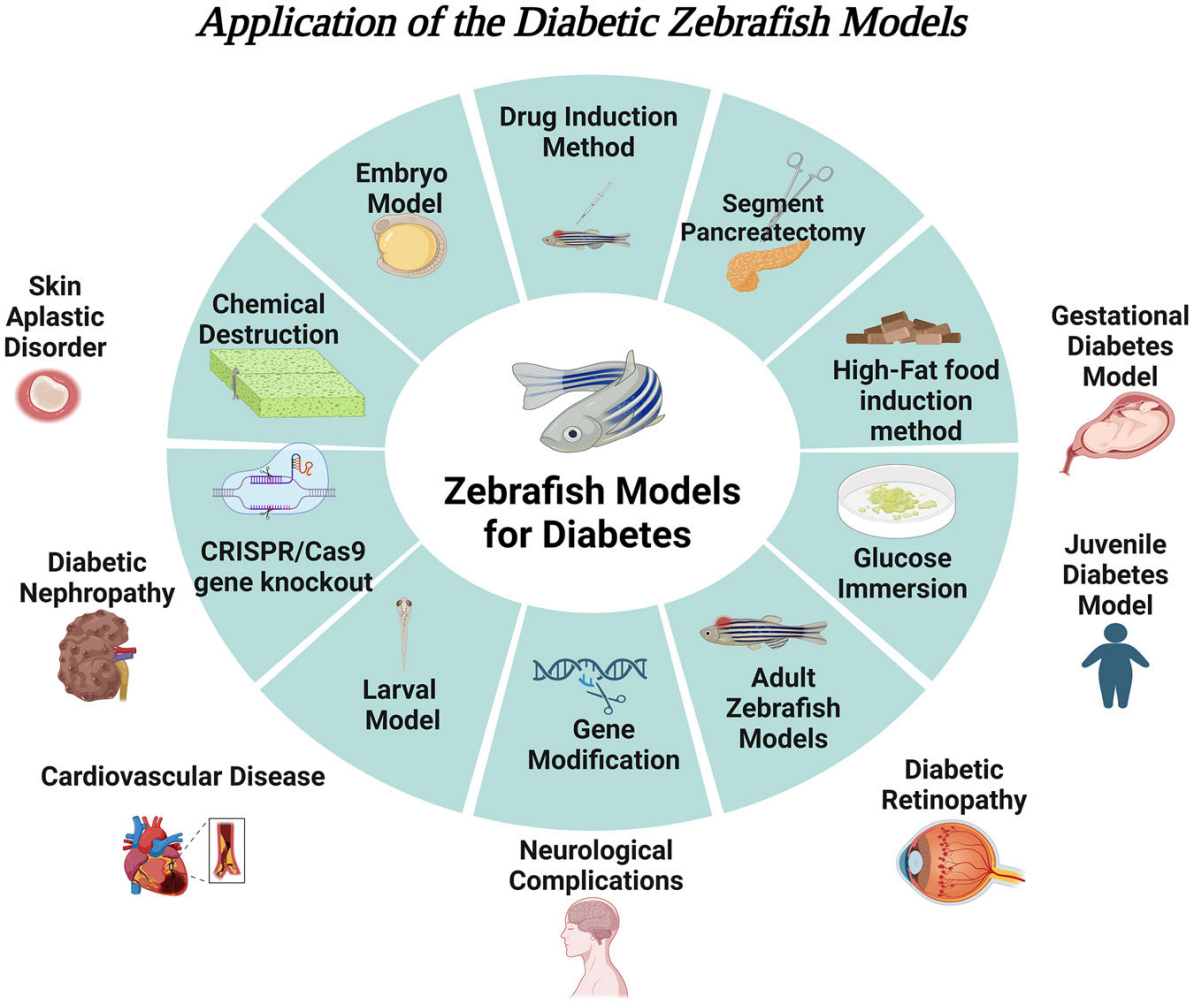
Various applications of zebrafish in different models for treatment of diabetes and its complications. Created with BioRender.com.

The earliest diabetic zebrafish models were created through chemical induction, dietary changes and surgical procedures, and with the advent of genetic tools, novel zebrafish models with mutant genes or transgenes producing fluorescent proteins have widely emerged. The combination of genetic with chemical or dietary approaches allows for the development of even more sophisticated zebrafish models (Figure 2) (Seth et al., 2013). Genetic methods are suggested as a more precise approach to target specific genes and generate more accurate and specific abnormal phenotypes compared to the other methods. Nonetheless, inducing diabetes through non‐genetic means may be a simpler approach and more accessible and time‐ and cost‐effective.

### 2.1 Chemical diabetic models

Chemical‐dependent β‐cell ablation models are the preferred method for modelling T1DM (Table 1). They are based on exposure to diabetogenic agents such as streptozotocin (STZ) and alloxan, which induce irreversible damage to pancreatic islets mediated by a mechanism driven by reactive oxygen species (Kulkarni et al., 2018). Bisphenol compounds, such as the commonly found compound bisphenol A and its alternatives bisphenol S, bisphenol F and bisphenol AF, have recently been shown to act as diabetogenic agents (Hong et al., 2023; Zhao et al., 2018). Their application in zebrafish models, on the other hand, However, their research in zebrafish models have not been related to diabetes or diabetes‐associated complications, in terms of pathophysiology or drug discovery. Thus, we will concentrate on STZ and alloxan in this review.

**TABLE 1:** Zebrafish diabetic models based on chemical β‐cell ablation.

Model type	Diabetes type	Zebrafish age	Treatment	Phenotype	Advantages	Disadvantages	Reference
STZ injection	T1DM	Adult (<1 year)	STZ (0.3% – 350 mg/kg) i.p. injection or caudal fin injection (amputation)	Hyperglycaemia/retinopathy/nephropathy/impaired fin regeneration	Low cost/simple/novel experimental model for the study of regeneration in a diabetic background	Weekly injections (3 weeks) to induce prolonged state of very high hyperglycaemia/5% death expected during induction period	Olsen et al., 2010
	T1DM	Adult (<1 year)	STZ (0.3% – 350 mg/kg) i.p. injection	Hyperglycaemia/retinopathy/nephropathy/impaired fin regeneration	Short time to complete an experiment from diabetes induction until metabolic memory examination (80 days)/ease of approaching caudal fin regeneration/simple and quantifiable method to assess metabolic memory	No major difficulties reported	Intine et al., 2013
	T1DM	Adult (<1 year)	STZ (0.3% – 350 mg/kg) i.p. injection	Hyperglycaemia/retinopathy/nephropathy/impaired fin regeneration	Allows analysis of molecular aspects of the DM and metabolic memory states in ways that cannot be experimentally approached with traditional diabetic models	No major difficulties reported	Sarras et al., 2013
	T1DM	Larvae (age n.s.)	STZ (saturated solution; exact doses n.s.) pericardial injection	Hyperglycaemia/Abnormal capacity for fin regeneration	Experimental model in larvae for transient hyperglycaemia due to β‐cell apoptosis/larval anatomical advantages	Larvae's small size and insufficient volume of plasma/repetitive microinjections (three times every 3 days)	Wang et al., 2020
	T1DM	Adult (<1 year)	STZ (7% – 350 mg/kg) i.p. injection followed by STZ (7%) intravitreal injection	Hyperglycaemia/retinopathy	Low cost/simple/high‐throughput platform for screening chemicals affecting Glu metabolism	No major difficulties reported	Wang et al., 2020
AX exposure	T1DM	Adult (age n.s.)	AX (0.3%) exposure for 30 min, followed by aqueous glucose solution (1%) for 30 min, and water for 1 h	Acute hyperglycaemia (destroyed pancreas)	Low‐cost alternative T1DM animal model and no death occurred during the induction process	Successive induction steps	Ighodaro et al., 2017
	T1DM	Larvae (5 dpf)	AX (600 μM) exposure for 72 h	Hyperglycaemia/diabetic hearing loss	Low cost/simple methodology	No major difficulties reported	Shin et al., 2012
	T1DM	Larvae (5 dpf)	AX (300 μM) exposure for 72 h	Hyperglycaemia/sensorineural damage	Simple and validated diabetic neuromast larval zebrafish model that allowed for the study of diabetic sensorineural damage and for testing drugs	Alloxan toxicity (zebrafish had significantly decreased heartbeat and decreased hatching rate)	Nam et al., 2019
	T1DM	Larvae (5 dpf)	AX (100 μM) exposure for 72 h	Hyperglycaemia/pancreatic islets significantly decreased	Simple and validated model for testing drugs to restore alloxan‐induced pancreatic islet damage	Alloxan toxicity (zebrafish had significantly decreased heartbeat and decreased hatching rate)	Nam et al., 2019

Abbreviations: AX, alloxan monohydrate; DM, diabetes mellitus; dpf, days post‐fertilization; Glu, glucose; n.s., not specified; STZ, streptozotocin; T1DM, type 1 diabetes mellitus; T2DM, type 2 diabetes mellitus; WT, wild type.

#### 2.1.1 STZ injection

STZ is the most used chemical‐induction model to attain diabetic zebrafish with the advantages of being a relatively simple, low‐cost and precise approach that is selectively toxic to pancreatic β‐cells. One of the first studies to use STZ generated a drug‐induced zebrafish model of T1DM that resulted in sustained hyperglycaemia with reduced insulin secretion by the endocrine pancreas and early signs of retinopathy and nephropathy (Table 1) (Olsen et al., 2010). STZ was administered in adult zebrafish by three alternating i.p. injections during the first week, resulting in a rapid increase in the levels of fasting blood glucose. If zebrafish received one weekly maintenance injection, hyperglycaemia was maintained throughout the time, and normoglycaemia was restored after STZ administration was halted. In contrast, caudal fin regeneration remained impeded to the same extent as during the acute hyperglycaemic condition, indicating the presence of metabolic memory. This was the first study to characterize a reduction in the regenerative capacity of STZ‐induced diabetic zebrafish, comparable to the difficulty of wound healing in humans, which made it a suitable model for studying the regeneration process in the presence of diabetic pathology. Furthermore, the hyperglycaemic showed a substantially thicker glomerular basement membrane, while the eyes had a thinner inner plexiform layer and photoreceptor segment layer, which enabled the authors to propose the zebrafish model for studying diabetic nephropathy and retinopathy (Intine et al., 2013). A few years later, Intine et al. (2013) confirmed fin regeneration impaired after STZ removal was due to a metabolic memory phenomenon by using the same STZ‐induced model described by Olsen et al. (2010). This was a novel model that allowed for the examination of the purely epigenetic components that support metabolic memory. Within the same research group, further investigations using the same STZ‐induced T1DM model helped to bring insights into the metabolic memory mechanism of diabetic zebrafish (Sarras et al., 2013). These findings indicated that the regeneration pathways and genes in zebrafish were shared across a wide range of tissue types, including the fin (a connective tissue structure), retina (a central nervous system structure) and heart (a muscle structure). This was significant in terms of regenerative medicine since it allowed these discoveries to potentially be extrapolated to the more fundamental mechanism of zebrafish tissue regeneration in general.

Profiting from the protruding cardiac region and transparent body that facilitate real time imaging visualization, a more recent study developed a larval STZ‐induced ablation model, which was attained by direct pericardial STZ injection under a microscope (Wang, Yang et al., 2020). STZ delivery into zebrafish larvae caused β‐cell apoptosis and lack of insulin, followed by glucose metabolic and fin regeneration abnormalities. The fin regeneration was transiently impaired, and its gradual recovery and acceleration corresponded to the gradual increase in insulin accumulation due to the regeneration of β‐cells, and, in turn, to normoglycaemia reestablishment (Wang, Yang et al., 2020). Moreover, an STZ‐induced retinopathy model in adult zebrafish was attained by a single i.p. STZ injection, and the acceleration of retinopathy was made on 7‐day diabetic zebrafish by an intravitreal STZ injection (Wang, Du et al., 2020). As a result, zebrafish showed increased levels of glucose and several diabetes‐associated vascular complications, including increased levels of homocysteine, eye and zebrafish body weight ratio, tissue thiobarbituric reactive substances, and arginase reductase activity, reduced glutathione (GSH) and changes of visual functions.

#### 2.1.2 Alloxan exposure

Alloxan is significantly less expensive and more widely available than streptozotocin, but it is less often utilized owing to various drawbacks. It is a very unstable chemical that rapidly undergoes redox cycling and generates unstable hyperglycaemia characterized by a multiphasic blood glucose response, making use of potential anti‐diabetic or hypoglycaemic drugs challenging (Ighodaro et al., 2017). Even in the few cases where apparent stability is obtained, the typical duration of such stable hyperglycaemia is less than a month, which is insufficient for proper evaluation of a test drug. One of the first studies to develop an alloxan‐induced zebrafish model of T1DM resulted in acute hyperglycaemia with a destroyed pancreas (Shin et al., 2012). It consisted of treating adult fish with a 300 mg/100 mL solution for 30 min, with subsequent induction steps (Table 1). Despite being proposed as an alternative hyperglycaemia animal model, to our knowledge, it was not further applied, possibly because it allowed only for short‐term experimentation and because of the successive induction steps it demanded. Thereafter, a novel pharmacological alloxan‐induced model for auditory regeneration and protection was reported in larval zebrafish, which developed T1DM with hearing loss. Zebrafish larvae were exposed to alloxan for 72 h developed hearing loss, which was recovered after treatment with trigonelline, as explained further.

This same approach was further applied to develop an alloxan‐induced diabetic neuromast larval zebrafish model that allowed for the study of diabetic sensorineural damage, a complication of the sensory neural system resulting from long‐term hyperglycaemia (Nam et al., 2019). In this case, a natural extract, Korean red ginseng, was tested for its capacity to recover diabetic neuromast zebrafish, which was attained, and the extract was suggested to promote the nerve growth factor pathway. A recent study published by the same research group reported a modification in the protocol; they induced pancreatic islet damage in zebrafish larvae via the administration of 100 μM alloxan for 15 min, followed by replacement with a 0.03% sea salt solution. Alloxan significantly decreased the size of pancreatic islets as well as glucose uptake in zebrafish, and the administration of a well‐known antidiabetic drug could reverse such negative effects induced by alloxan, thus validating the model for drug testing.

### 2.2 Dietary and insulin induction zebrafish models

When conducting diabetes research using zebrafish models, it is essential to consider their distinctive dietary composition. Standard zebrafish feed typically contains higher protein levels and lower carbohydrates compared to the human diet (Gleeson et al., 2007). This composition affects insulin's role in glucose regulation, protein synthesis and amino acid uptake (Dorsemans et al., 2017; Mohammadi et al., 2020). The higher protein content in zebrafish diets may influence the balance between glucose regulation and amino acid metabolism, as insulin‐mediated pathways in zebrafish might resemble those in carnivorous mammals with high‐protein, low‐carbohydrate diets. Therefore, researchers should account for these dietary differences to ensure the generalizability of their findings to human diabetes. Further studies on the impacts of zebrafish diet on their metabolic processes can provide insights into the suitability and limitations of these models for studying human diabetic conditions. This could lead to refined zebrafish models that better mimic human metabolic conditions, enhancing the translational value of research findings in the context of diabetes and its treatment.

#### 2.2.1 High glucose treatment

Direct immersion of zebrafish in a high glucose solution is the easiest and fastest way to induce hyperglycaemia and diabetic symptoms and may be the most straightforward way to establish a T2DM diabetic model. This model encompasses a diverse range of relatively simple and cost‐effective approaches, such as exposure to high fluctuating glucose concentrations (Gleeson et al., 2007), constant concentrations (Dorsemans et al., 2017) and stepwise increasing concentrations (Mohammadi et al., 2020). These models allowed the advancement of the study of diabetes and diabetes complications as elevated blood glucose could be maintained in adult zebrafish for a longer period, resembling human T2DM (Table 2).

**TABLE 2:** Zebrafish diabetic models based on dietary and insulin induction approaches with glucose immersion.

	Method	Model type	Diabetes type	Zebrafish age	Treatment (chemical or feeding)	Phenotype	Advantages	Disadvantages	Reference
Dietary and Insulin Induction	Glucose immersion	Fluctuating dosis	T2DM	Adult (>1 year old)	Oscillating immersion between Glu 2% and 0% solutions every 24 h during 30 days	Hyperglycaemia/retinopathy	Simple experimental approach to study retinopathy	High variability in blood glucose levels between fish in the same experimental group	Gleeson et al., 2007
		Constant dosis	T2DM	Larvae (4 dpf)	Transdermally treated with 25% Glu in Me_2_SO	Hyperglycaemia	Simple and drug‐validated larval model for the examination of Glu metabolism, using PEPCK as an indicator of blood glucose levels	No major difficulties reported	Dorsemans et al., 2017
			T2DM	Adult (age n.s.)	Chronic immersion in 111 mM Glu for 14 days	Hyperglycaemia/hyperinsulinaemia	Simple and drug‐validated experimental approach	20% mortality (similar between treated and control fish)	Dorsemans et al., 2017
			T2DM	Adult (<1 year old)	Chronic immersion in 111 mM Glu for 14 days	Hyperglycaemia/brain dysfunction	Robust model for studying the effects of metabolic disorders on the CNS	No major difficulties reported	Mohammadi et al., 2020
		Stepwise increasing dosis	T2DM	Adult (<1 year old)	1% Glu for 2 weeks, 2% Glu for 2 weeks and 3% Glu for 4 weeks	Hyperglycaemia	Simple experimental approach to examine how prolonged hyperglycaemia impacts various organ systems	No major difficulties reported	Mohammadi et al., 2020
			T2DM	Adult (<1 year old)	1% Glu for 2 weeks, 2% Glu for 2 weeks and 3% Glu for 4 weeks	Hyperglycaemia/visual and cognitive deficits	Simple experimental model to study the association between hyperglycaemia, learning and recovery	No major difficulties reported	Li et al., 2017
			T2DM	Adult (<1 year old)	1% Glu for 2 weeks, 2% Glu for 2 weeks and 3% Glu for 4 weeks	Hyperglycaemia/visual and cognitive deficits	Further studied hyperglycaemia‐induced cognitive deficits	No major difficulties reported	Mohammadi et al., 2020
			T2DM	Adult (<1 year old)	50 mM Glu for 4 days, 100 mM for 3 days and 200 mM Glu for 13 days	Hyperglycaemia/insulin resistance/FBG/altered inflammation markers and liver enzymes	Rapid, effective and drug‐validated model for the early phase of T2DM research	No major difficulties reported	Dorsemans et al., 2017
	High fat diet treatment	HFD	T2DM	Adult (age n.s.)	Brine shrimp (60 mg cysts per fish daily) plus 1% egg yolk twice daily	Hyperglycaemia/insulin resistance	Low cost/simple methodology	Long time of induction (10 weeks)	Dorsemans et al., 2017
		HFD + HG	T2DM	Adult (age n.s.)	Small‐fish special food with cholesterol (10%) + Glu (2%) twice daily	Hyperglycaemia/robust anxiety‐like behaviour	Model for DM‐related affective pathogenesis	Long time of induction (19 days)/did not assess the effect of HFD and HG individually	Wang et al., 2020
		HFD + HG	T2DM	Adult (<1 year)	HG (50 mM)	Hyperglycaemia/inflammatory milieu/insulin resistance/impaired β‐cells	Experimental diabetic model to study histopathological changes in liver and intestines	Could not directly measure insulin resistance as well as the oxidative stress process	Mohammadi et al., 2021
					HFD (ART six times/day)				
					HG (50 mM) + HFD (ART six times/day)				
	Insulin administration	Injection	T2DM	Larvae (4 dpf)	Injection in caudal aorta (dosis n.s.)	Hyperinsulinaemia/hyperglycaemia/immune suppression	New tool for the study of insulin metabolism and insulin resistance study in a non‐obese state	No major difficulties reported	Marin‐Juez et al., 2014
		Exposure	T2DM	Larvae (3 dpf)	Treatment with human insulin (10 μM) for 48 h	Hyperinsulinaemia/hyperglycaemia/damaged pancreatic islets and β‐cells	Rapid insulin‐resistant zebrafish model that complements the existing rodent models, validated for drug testing and drug discovery	No major difficulties reported	Nam et al., 2021
			T2DM	Larvae (3 dpf)	Treatment with insulin (250 nM) and reinduced after 24 h (100 nM)	Hyperglycaemia/hyperinsulinaemia/BBB injured	Robust and less invasive T2DM and hyperinsulinaemia model in larvae, allows unravelling of the mechanism associated with neurodegeneration and phenotype‐driven drug discovery against diabetes	No major difficulties reported	Md Razip et al., 2022

Abbreviations: ART, *Artemia*; BBB, blood–brain barrier; CNS, central nervous system; DEX, dexamethasone; DM, diabetes mellitus; dpf, days post‐fertilization; FBG, fasting blood glucose; Glu, glucose; HBG, high blood glucose; HCD, high cholesterol diet; HFD, high fat diet; HG, high glucose; n.s., not specified; PEPCK, phosphoenolpyruvate carboxykinase; T1DM, type 1 diabetes mellitus; T2DM, type 2 diabetes mellitus; WT, wild type.

One of the first reports of a viable zebrafish diabetic model was by Gleeson et al. (2007), who induced hyperglycaemia in adult zebrafish by alternately immersing the fish in 2% glucose solution or water every 24 h. As a result, zebrafish developed retinopathy and both the inner plexiform layer and the inner nuclear layer were significantly reduced in the retinas of treated fish compared to untreated fish, similar to that seen in other animal models of diabetes and diabetic patients. The model developed by Elo et al. (2007) consisted of treating adult zebrafish transdermally with 25% glucose in dimethyl sulfoxide. The importance of this approach was that the authors successfully made up for the incapacity to accurately measure blood glucose levels in larvae by analysing phosphoenolpyruvate carboxykinase (PEPCK) expression, with the extra advantage that its regulation is similar to that in humans and mice (Gleeson et al., 2007). The PEPCK enzyme catalyses a rate‐limiting step in gluconeogenesis and is transcriptionally regulated by glucagon and insulin; thus, its expression can act as a sensitive marker of blood glucose levels. A simpler and more effective T2DM model in adult zebrafish was attained by immersion in a 111 mM glucose solution for 14 days (Li et al., 2017). Diabetic zebrafish developed increase glycation of eye proteins, decrease mRNA levels of insulin receptors in muscle, high blood glucose and impaired response to exogenous insulin. Overall, the authors were able to confirm that the exposure of adult zebrafish to high and constant glucose concentration led to persistent metabolic changes probably underlain by a hyperinsulinaemic state and peripheral impaired glucose metabolism.

One of the first studies to investigate the effect of hyperglycaemia on brain homeostasis and neurogenesis, on the other hand, used the well‐described hyperglycaemic zebrafish model (Dorsemans et al., 2017). The findings provided new evidence highlighting the evolutionarily conserved negative effects of hyperglycaemia on neurogenesis and brain healing in zebrafish, including impairment of the expression of genes involved in the establishment of the blood–brain barrier (BBB), claudin 5a, zona occludens 1a and b, as well as decreased brain cell proliferation in most neurogenic niches throughout the forebrain and the midbrain (Dorsemans et al., 2017; Ma et al., 2017; Mohammadi et al., 2020). Claudin 5 is a key component of the BBB that constitutes the basic framework to block unwanted molecules passing between blood and brain (Ma et al., 2017 and Li et al., 2017). Altogether, these results allowed us to reinforce the utility of zebrafish as a robust model for studying the effects of metabolic disorders on the central nervous system.

The same group further dug deeper into how hyperglycaemia induced cognitive deficits and showed it triggered a strong inflammatory response causing initial trending changes in tight junction and neuronal markers; increased glial fibrillary acidic protein and increased nuclear factor‐κB levels were detected in retina, as well as decreased physiological responses (Rowe et al., 2022, 2021). Another study approached hyperglycaemia and insulin resistance by a stepwise increasing glucose model in adult zebrafish, which allowed investigation of the inflammation, toxicity and gene expression resulting fromT2DM, as well as the capacity of drugs to ameliorate diabetic complications.

#### 2.2.2 High‐fat treatment

Because hyperlipidaemia frequently occurs along with hyperglycaemia in T2DM, high‐glucose (HG) and high‐fat diets (HFD) have been used for modelling diabetes and diabetic pathologies in zebrafish. For instance, adult zebrafish fed with a long‐term HFD developed T2DM and reported higher body weight and body mass index, more lipid vacuoles in the liver, increased insulin transcription levels in the liver, brain and muscle, and high fasting blood glucose, whereas insulin signalling pathway and glucose transport were impaired in the insulin‐targeting tissues (Table 2) (Meng et al., 2017). Altogether these changes may lead to dysregulation of cell homeostasis, which may be one of the factors involved in insulin‐resistance in T2DM.

Another study using a zebrafish model of HG/HFD revealed a connection between diabetes and affective pathogenesis (Wang, Wei et al., 2020, Wang, Li et al., 2020, Wang, Du et al., 2020, Wang, Yang et al., 2020). Zebrafish presented hyperglycaemia and hyperlipidaemia (high levels of triglycerides, total cholesterol and low‐density lipoprotein) and showed anxiety‐like behaviour, supported by elevated whole‐body cortisol and the cytokines interferon‐γ and interleukin‐4, as well as higher brain mRNA expression of the glucocorticoid receptor, the microglial biomarker CD11b, pro‐inflammatory cytokines interleukin‐6 and tumour necrosis factor‐α, the astrocytal biomarker glial fibrillary acidic protein, the neurotrophin brain‐derived neurotrophic factor, its receptors p75 and TrkB, and apoptotic Bax and caspase‐3. These findings supported the overlapping nature of diabetes‐related affective pathogenesis and emphasized the role of peripheral and central inflammation and apoptosis in diabetes‐related affective and neuroendocrine deficits in zebrafish.

Finally, a recent study shed light on the differential effects caused by the administration of HFD, HG and HG+HFD in adult zebrafish (Mohammadi et al., 2021). Diabetes induction with overfeeding and excessive glucose consumption is accompanied by higher expression of inflammatory cytokine genes in the pancreas and lower insulin production and GLU2 gene expression, which could be due to more β‐cell dysfunction in response to the inflammatory milieu. The highest rate of mortality occurred in the HFD+HG group (*Artemia* + glucose), possibly due to the presence of a higher inflammatory milieu and more severe major organ damage.

#### 2.2.3 Insulin administration

One of the first studies to develop a hyperinsulinaemia model injected human recombinant insulin into the caudal aorta of zebrafish larvae using a glass capillary (Marin‐Juez et al., 2014) (Table 2). The downregulation of the marker gene phosphoenolpyruvate carboxykinase 1 (*pck1*) after insulin injection led to the model's validation as its expression level is well known to be inhibited by insulin. Moreover, the downregulation of insulin metabolism was confirmed by the downregulation of genes known to play a central role in this pathway (*insr*, *irs1*, *irs2*, *pik3cb*), and an important connection was proposed between insulin regulation and the immune system through tyrosine‐protein phosphatase non‐receptor type 6 (PTPN6) as a key mediator. The authors showed the suitability of the model for the potential discovery of novel regulators of insulin resistance and its usefulness for studies of the function of immunological determinants in a non‐obese model system.

A simpler insulin‐resistance approach was further developed based on incubating zebrafish larvae in a high‐insulin solution (10 μM) for 48 h, rather than injecting insulin (Nam et al., 2021). It resulted in hyperinsulinaemic conditions that led to increased levels of fasting blood glucose, decreased glucose tolerance and pancreatic islet cell damage. Insulin resistance altered gene expression due to the mitogen‐activated protein kinase (MAPK) and calcium signalling pathways. Thereafter, the study approached an insulin‐resistance model by exposing zebrafish larvae to insulin at the concentration previously validated by Marin‐Juez et al. (2014). Larvae developed hyperglycaemia, and the hyperinsulinaemic state was confirmed as altered insulin signalling resulted. The expression of the marker genes *pepck* and *akt* was downregulated. The *akt* gene is known to be a central component in the signalling pathways activating the kinase family for assessing early‐stage insulin resistance. Moreover, zebrafish larvae also developed an oxidative stress confirmed by the substantially high levels of the biomarker malondialdehyde (MDA), and the upregulation of MAPK and claudin‐5a genes. Altogether, these results confirm that the mechanisms of neurogeneration were altered and the blood–brain barrier further injured (Md Razip et al., 2022).

### 2.3 Genetic zebrafish models

In 2007, scientists began using genetic models of zebrafish to investigate diabetes. Genetic zebrafish models can be obtained using a variety of methods, ranging from basic expression of the *Escherichia coli nfsB* gene under the insulin promoter to cutting‐edge technologies such as the CRISPR/Cas9 gene editing system. Such genetic technologies may be supplemented with chemical or dietary induction methods or even induced by hypoxia to better reproduce diabetes or diabetic diseases in transgenic or mutant lines. Furthermore, transgenic zebrafish expressing any fluorescent protein, together with advances in imaging techniques, enable non‐invasive examination and quantification of nearly any zebrafish organ. Following that, we present the most important genetic models that have been supplemented by induction with high‐glucose or chemical approaches (Table 3) (Delaspre et al., 2015). By employing molecular techniques to ablate pancreatic β‐cells, researchers were successful in inducing a diabetic state in the fish. Subsequently, during the regenerative phase, the β‐cells re‐formed due to the fish's capacity to regenerate lost tissue. The ablation was achieved by expressing a nitroreductase enzyme, which transforms prodrugs into poisonous substances. The researchers visualized the β‐cells by fusing the enzyme with a fluorescent protein. It was observed that only the β‐cells were impacted by the treatment, while other cells in the pancreatic islets remained unscathed. After the treatment was discontinued, the β‐cells regenerated within 36 h. This model allowed researchers to investigate β‐cell regeneration and identify factors that promote or inhibit the process. They utilized cytomarkers and bromodeoxyuridine incorporation to monitor cell lineage and proliferation.

**TABLE 3:** Zebrafish diabetic models based on genetic approaches.

Method	Model type	Diabetes type	Zebrafish age	Transgene/genotype	Treatment	Phenotype	Advantages	Disadvantages	Reference
NTR/MTZ β‐cell ablation	*Tg(T2Kins:nfsBmCherry)^jh4^*	T1DM	Larvae (56 hpf)	Expression of *nfsB* under insulin promoter *ins* (β‐cell ablation line)	MTZ (5 or 10 mM) treatment	Hyperglycaemia/NTR‐induced specific ablation of pancreatic β‐cells (destroyed islet tissue)/increased free glucose levels	Prodrug dependent system allowing for spatial and temporal control of cell ablation, with minimal disturbance to the tissue environment.	No major difficulties reported	Delaspre et al., 2015
	*Tg(cmlc2:CFP‐NTR)^s890^*	T1DM	Embryos, larvae, juvenile and adult	Expression of *nfsB* under cardiomyocyte promoter *cmlc2*	MTZ (5 or 10 mM) treatment	Cardiomyocytes die/heart stops contracting	Great flexibility regarding the target tissue to be ablated/allows for the elucidation of cellular and molecular mechanisms underlying tissue regeneration after ablation	The ability to ablate certain cell populations or tissues depends on the availability of an appropriate promoter to drive NTR expression (e.g., hepatocytes)	Md Razip et al., 2022
	*Tg(fabp10:CFP‐NTR)^s891^*			Expression of *nfsB* under hepatocyte promoter *fabp10*		Significant apoptosis in hepatocytes			
	*Tg(ins: CFP‐NTR)^s892^*			Expression of *nfsB* tagged to CFP protein under insulin promoter *ins*		Hyperglycaemia/β‐cell reduction or complete ablation			
	*Tg(T2Kins:nfsBmCherry)^jh4^*	T1DM	Adult (<1 year)	β‐Cell ablation line	MTZ (30 mM) injected intracelomically	Hyperglycaemia/destroyed islet tissue/increased free glucose levels	β‐Cell regeneration model to study mechanisms to improve regeneration in diabetic patients	No major difficulties reported	Delaspre et al., 2015
	*Tg(ins:Flag‐NTR)^s950^*	T1DM	Larvae (106 hpf)	β‐Cell ablation line	MTZ (2.5 or 7.5 mM) treatment	Hyperglycaemia/oxidative stress in ablated β‐cells	Robust model to monitor ROS dynamics in β‐cells	No major difficulties reported	Delaspre et al., 2015
	*Tg(nkx2.2a:megfp; ins:nfsB‐mcherry)*	T1DM	Larvae (5 dpf)	Double transgenic line expressing *nkx2.2a+* (motor nerve‐associated perineurial glia) tagged to *mEGFP* in β‐cell ablation line	MTZ (10 mM)	Hyperglycaemia/β‐cell full ablation/sensory and motor nerve components dysregulated	Novel DPN model that may help direct therapeutic approaches	No major difficulties reported	Rocker et al., 2019
	*Tg(NBT: DsRed; ins:nfsB‐mcherry)*			Double transgenic line expressing *nbt+* (motor neurons/axons) in β‐cell ablation line					
	*Tg(mbp:EGFP‐CAAX; ins:nfsB‐mcherry)*			Double transgenic line expressing *mbp* (myelin basic protein) tagged to EGFP in β‐cell ablation line					
	*Tg(neurod:egfp; ins:nfsB‐mCherry)*			Double transgenic line expressing *neurod+* sensory neurons tagged to EGFP in β‐cell ablation line					
	*Tg(ins: CFP‐NTR)^cq29^*	T1DM	Larvae (age n.s.)	Expression of the CFP–NTR fusion protein under insulin promoter	MTZ (10 mM) treatment	Nearly all the β‐cells ablated		No major difficulties reported	Wang et al., 2020
	*Tg(ins: mCherry‐NTR; pdx1:GFP)*			*Tg(pdx1:GFP)* transgenic background was applied in the β‐cell ablation line *Tg(ins: mCherry‐NTR)^cq30^*		Green fluorescent β‐cells emerging from pre‐existing *pdx1+* cells	A set of real‐time imaging of regenerating β‐cell in the primary islet after MTZ treatment		
	*Tg(ins: CFP‐NTR; mnx1:GFP)*			*Tg(ins: CFP‐NTR)* was used to label β‐cell blue with CFP and perform MTZ‐induced ablation; the *mnx1* specific β‐cell development regulator was marked green with GFP		Green fluorescent β‐cells emerging from pre‐existing *mnx1+* cells			
	*Tg(ins: CFP‐NTR; nkx2.2a:GFP)*			*Tg(ins: CFP‐NTR)* was used to label β‐cells blue with CFP and perform MTZ‐induced ablation; the *nkx2.2a* specific β‐cell development regulator was marked green with GFP		Green fluorescent β‐cells emerging from pre‐existing *nkx2.2a+* cells			
	*Tg(pdx1:GFP; ins: CFP‐NTR)*			β‐Cells stained blue, performed MTZ‐induced ablation, and the *pdx1* specific β‐cell development regulator was marked green with GFP		These transgenic lines allowed determination of the different cellular origins of β‐cell regeneration	These transgenic lines allowed determine of the different cellular origins of β‐cell regeneration		
	*Tg(pdx1:GFP; mnx1:GFP; ins: CFP‐NTR)*			β‐Cells stained blue, performed MTZ‐induced ablation, and *pdx1* and *mnx1* were marked green with GFP					
	*Tg(pdx1:GFP; nkx2.2a:GFP; ins: CFP‐NTR)*			β‐Cells stained blue, performed MTZ‐induced ablation, and *pdx1* and *nkx2.2a* were marked green with GFP					
	*Tg(mnx1:GFP; nkx2.2a:GFP; ins: CFP‐NTR)*			β‐Cells stained blue, performed MTZ‐induced ablation, and *mnx1* and *nkx2.2a* were marked green with GFP					
	*Tg(ins:loxP‐CFPNTR‐stoploxP‐DsRed)^cq31^*			Cre/loxP‐mediated lineage		Allowed for tracing of pancreatic ductal cells in β‐cell regeneration			
Pancreatectomy	*Tg(−1.0ins:EGFP)^sc1^*	T1DM	Adult (<1 year)	Expression of *EGFP* under insulin promoter	Physical removal of the GFP‐positive main pancreas	Hyperglycaemia	The model can be used to define pathways for islet‐cell regeneration in humans	Technically difficult/need to monitor for rapid gill movement or haemorrhage after surgery	Delaspre et al., 2015
Dietary Induction	*Tg(fli1:EGFP)y*	T2DM	Adult (1 and 2 years)	Expression of *EGFP* in the entire vasculature under the control of the *fli1* promoter, enabling the visualization of vascular defects in live zebrafish embryos	Fluctuating Glu (2% and 0% solutions) every 24 h during 30 days	Hyperglycaemia/vascular changes (thickening of the basal membrane, disturbance of the retinal blood barrier and dilatation of retinal capillaries)/neurodegeneration of cone photoreceptors without drastic effects on other retinal cell types	Novel model of non‐proliferative diabetic retinopathy	Long time of induction (30 days)	Alvarez et al., 2010
	*Tg(fli1:egfp)*	T2DM	Embryos (3 dpf)	Expression of *EGFP* in the entire vasculature under the control of the *fli1* promoter (Friend leukaemia integration 1 transcription factor)	Fluctuating Glu (0%, 4% and 5%) by pulsative application every 24 h up to 5 dpf	Numerous fetus ocular defects (altered retinal cell layer thickness, increased presence of macrophages, and decreased number of Müller glial and retinal ganglion cells)	Innovative gestational hyperglycaemia model to mimic the fluctuations in glycaemia experienced by the developing fetus in pregnant women with DM/legacy of HG exposure may extend into adulthood	No major difficulties reported	Wen et al., 2021
	*Tg(shh:gfp)*			Expression of *GFP* under *shh* (sonic hedgehog signalling molecule)					
	*Tg(gfap:GFP)*			Expression of *GFP* under *gfap* (glial fibrillary acidic protein)					
	*Tg(mpeg:dsRed)*			Expression of *dsRed* under macrophage expressed gene					
	*Tg(fli1:egfp) × Tg(mpeg:dsRed)*			Double transgenic line expressing *EGFP* under *fli1* and macrophage expressed gene under *dsRed*					
	*Tg*(*flk*:EGFP)	T2DM	Larvae (3 dpf)	Expression of *EGFP* under the regulatory sequences of *flk1* (*Kdrl*) for vascular endothelial cells	Chronic Glu 130 mM for 3 days	Hyperglycaemia/retinopathy	Novel, short‐term, in vivo screening method for compounds affecting DR	No major difficulties reported	Xu & Wu, 2021
	*Tg(flk:EGFP)*	T2DM	Embryos (3–4 dpf)	Expression of *EGFP* under the regulatory sequences of *flk1* (*Kdrl*) for vascular endothelial cells	Chronic Glu 160 mM for 4 days	Hyperglycaemia/retinopathy	Reliable model for screening, comparing, and analysing changes leading to new drugs for preventing DR	No major difficulties reported	Wen et al., 2021
	*Tg*(*kdrl:GFP*)	T2DM	Adult (male; age n.s.)	Expression of *kdrl* fused to GFP as vascular endothelium reporter	Chronic in Glu 4% for 28 days and feeding three times/day with commercial food	Hyperglycaemia/osteoporotic‐like phenotype/retinal vasculopathy	Simple experimental approach to elucidate in vivo the molecular mechanisms of metabolic changes, which influence the bone tissues regulation in human diabetic patients, and for drug screening	Not applicable for female zebrafish	Carnovali et al., 2016
	*Tg(kdrl:Has.HRAS‐mCherry)*	T2DM	Larvae (8 dpf)	Labels endothelial cell membranes	Chronic Glu (20 mM) for 2, 60, 96 and 120 h	Hyperglycaemia/cerebrovascular patterning/neurovascular coupling	First non‐mammalian model of neurovascular coupling, offering the potential to perform more rapid genetic modifications and high‐throughput screening than is currently possible using rodents.	No major difficulties reported	Chhabria et al., 2020
	*Tg(gata1:DsRed)*			Labels erythrocytes allowing quantification of erythrocyte/RBC speed					
	*Tg(nbt:GCaMP3)*			Neuronal cytoplasmic Ca levels reported by *GCaMP3* under control of the neuronal beta‐tubulin (NBT) promoter					
	*claudin5a:GFP*			Expression of the transgenes *claudin5a:GFP* and *kdrl:mCherry* (reporter line)					
	*Tg(myl7:EGFP)*	T2DM	Larvae (3–9 dpf)	Expression of *EGFP* under the *myl7* promoter (cardiac myosin light chain 7)	Chronic Glu – STZ treatment – DM zebrafish treated with TER to induce HF	Hyperglycaemia/impaired glucose homeostasis/heart failure	Short induction time/can minimize differences among individuals because it does not require a diet/induces hyperglycaemia without destroying pancreatic β‐cells/non‐invasive	The DM‐HF larvae model must be confirmed in rodents and humans since zebrafish have different cardiac circulatory systems than rodents or humans	Kim et al., 2023
	*Tg (ins:EGFP)*			Expression of *EGFP* under the *ins* promoter (targeting pancreatic β‐cells)					
	*Tg(U6x:sgRNA(insra/b); LC) × Tg(actb2:cas9; LR)*	T2DM	Larvae (5 dpf)	Expression of *cas9* and sgRNA for tyrosinase *tyr*, and the insulin receptors *insra* and *insrb* loci mutation	HFD (5% egg yolk feeding)	Hyperglycaemia/visible pigmentation phenotype in the retinal pigment epithelium	Feasible, rapid, and straightforward technique/multiplex conditional mutagenesis can be achieved/breeding is reduced to one generation	No major difficulties reported	Cao et al., 2010; Cao et al., 2008
	*Tg(fabp10:cas9, CG) × Tg(U6x:sgRNA(insra/b); LC)*			Expression of *cas9* and sgRNA for biallelic gene inactivation of *insra* and *insrb* in somatic cells					
	*Tg(fli1:EGFP)*	T2DM	Larvae (age n.s.)	Expression of *EGFP* under the *fli1* promoter (a well described endothelial cell marker)	HCD (10%) + HG (3%)	Hyperglycaemia/increased tissue levels of insulin, glucagon, glucose/increased levels of total triglyceride and cholesterol/vascular abnormalities	Diabetes‐induced vascular changes occur in a relative short time period and can be quantified in living zebrafish	Zebrafish larvae are too small to acquire accurate biochemical estimations individually so their whole bodies need to be pooled	Wen et al., 2021
	*Tg(lys:EGFP)*			Expression of *EGFP* under the lysozyme C (*lysC*) promoter					
	*Tg(gata1:dsRed)*			Expression of *dsRed* under *gata1* promoter (an essential transcription factor for the erythroid cell lineage development in vertebrates)					
	*Tg(gata1:dsRed/fli1:EGFP)*			Double transgenic zebrafish expressing both *dsRed* and *EGFP*					
Overfeeding	InsGFP zebrafish: *Tg(−1.0ins:EGFP)^sc1^*	T2DM	Adult (male, <1 year)	Expression of *EGFP* in a dominant‐negative IGF‐I receptor (*IGF‐IR*) in skeletal muscle	Commercially available fish food using an automated feeding system (six feeds/day)	Hyperglycaemia/DIO/increased FBG level/insulin resistance/impaired Glu tolerance by overfeeding	Rapid onset of T2DM phenotypes compared with rodent models/pathological similarity in transcriptomic pathways to the human platform	Long time of induction (8 weeks)	Wongdee & Charoenphandhu, 2015
*Pdx1* mutant	*Tg*(*pdx1+/−;neuroD:EGFP)*	T2DM	Embryos, larvae and juvenile	Expression of *pdx1* (also known as insulin promoter factor 1, *ipf1*)	HFD (SDS100 commercial fry feed, 55% protein + 14% lipid)	Hyperglycaemia/retinopathy	*pdx1* mutant fish allows for in vivo monitoring of early stages of hyperglycaemia‐induced pathologies along the time/easy manipulation during larval stages	*pdx* null mutation reduced adult's body size and weight, and decreased viability	Wang et al., 2013
	*Tg(pdx*−/−)	T2DM	Larvae (6 dpf)/adult (<1 year)	CRISPR/Cas9‐mediated knockout of *pdx* gene in the transgenic lines *Tg(wt1b:EGFP)* and *Tg(fli1:EGFP)*	n.a.	Hyperglycaemia/retinopathy (extensive proangiogenic alterations in blood vessel morphology and metabolic alterations)	Novel model to study mechanisms of hyperglycaemia‐induced retinopathy wherein extensive proangiogenic alterations in blood vessel morphology and metabolic alterations underlie the vascular phenotype	Heavily impaired survival of *pdx1−/−* mutants into adulthood/not all vascular changes seen in DR are recapitulated by the phenotype	Ali et al., 2020
	*Tg(pdx*−/−)	T2DM	Larvae (1–2 dpf)/adult (<1 year)	CRISPR/Cas9‐mediated knockout of *pdx* gene in the transgenic lines *Tg(hb9:GFP), Tg(fli1:EGFP), Tg(nflk:EGFP)*, and *Tg(gata1a:DsRed)*	n.a.	Hyperglycaemia/nephropathy	This study shows for the first time that *pdx1* knockout leads to the development of early DN features in zebrafish and highlights the involvement of PtdE in the onset of DN	Heavily impaired survival of *pdx1−/−* mutants into adulthood	Kimmel et al., 2015
Hypoxia	*Tg(fli1:EGFP)*	T2DM	Adult (5–18 months)	Expression of *EGFP* in the entire vasculature under the control of the *fli1* promoter (Friend leukaemia integration 1 transcription factor)	N_2_‐induced hypoxia (the gas was perfused directly into the water to a final 10% air saturation over 48−72 h)	Retinopathy/retinal neovascularization	Non‐invasive and clinically relevant retinal angiogenesis model in adult zebrafish	No major difficulties reported	Cao et al., 2008

Abbreviations: CFP, cyan fluorescent protein; DIO, diet‐induced obesity; DM, diabetes mellitus; DM‐HFrEF, diabetes mellitus‐induced heart failure with reduced ejection fraction; DN, diabetic nephropathy; dpf, days post‐fertilization; DPN, diabetic peripheral neuropathy; DR, diabetic retinopathy; dsRed, red fluorescent protein; EGFP, enhanced green fluorescent protein; FBG, fasting blood glucose; Glu, glucose; HCD, high cholesterol diet; HF, heart failure; HG, high glucose; hpf, hours post‐fertilization; mEGFP, monomeric EGFP; MTZ, metronidazole; n.a., not applicable; n.s., not specified; NO, nitric oxide; NTR, nitroreductase; RBC, red blood cells; STZ, streptozotocin; T1DM, type 1 diabetes mellitus; T2DM, type 2 diabetes mellitus; VEGF, vascular endothelial growth factor; WT, wild type.

#### 2.3.1 Nitroreductase/metronidazole system

The nitroreductase (NTR)/metronidazole (MTZ) β‐cell ablation system was one of the first and most widely used hybrid genetic chemical techniques. It consists of transgenic zebrafish lines that express the *nfsB* gene, which encodes a bacterial nitroreductase (NTR) enzyme that converts the prodrug metronidazole (MTZ) to cytotoxins that induce specific cell death according to the promoter used. Many authors have used the NTR/MTZ system to study diabetes and diabetic pathologies through the ablation of pancreatic β‐cells when using the insulin promoter in the well‐known *Tg(ins:nfsB‐mcherry)* zebrafish line (abbreviated as InsNTR) (Delaspre et al., 2015) (Table 3). Although β‐cell apoptosis occurs rapidly at 18–24 h after MTZ treatment, the ablation model is hampered by the fact that zebrafish have a remarkable regeneration potential, with β‐cell mass being restored within 3–4 days of MTZ removal. Nonetheless, one of the main advantages of this model are its great flexibility regarding the target tissue to be ablated and that it can be used in wild‐type, mutant or genetically modified backgrounds. Thus, the expression of the *nfsB* gene under almost any promoter has resulted in new transgenic lines that have helped to expand the boundaries of knowledge of diabetes and diabetic complication pathophysiology. For example, using this kind of hybrid chemical genetic approach using the transgenic nitroreductase‐expressing zebrafish line *Tg(ins:Flag‐NTR)^s950^*, it was demonstrated that reactive oxygen species (ROS) were rapidly generated in β‐cells (Kulkarni et al., 2018), and a ROS‐based model was proposed for the mechanism of NTR/MTZ action in transgenic eukaryotic cells where ROS can be generated in a β‐cell‐specific manner.

Another study crossed the InsNTR zebrafish line with diverse transgenic lines (specified in Table 3) that allowed study of the effect of hyperglycaemia in zebrafish larvae on several components of the peripheral nervous system (Rocker et al., 2019). They found a rapid onset of dysregulation of several peripheral nervous system components in an acute hyperglycaemic period; *neurod* positive sensory neurons were misorganized concerning their normal confinement to the dorsal root ganglia, fewer motor nerves were properly ensheathed by *nkx2.2a^+^* perineurial glia, and tight junctions along the peripheral motor nerve were regularly absent/attenuated (Rocker et al., 2019). This was a novel study that allowed determination of the molecular mechanisms underlying diabetic peripheral neuropathy. Moreover, a recent study has developed a zebrafish β‐cell ablation and regeneration model to investigate β‐cell neogenesis in the first few days after a near‐total β‐cell loss^47^ (Wang, Wei et al., 2020). The combination of the NTR/MTZ approach with novel transgenic backgrounds labelling subpopulations of pancreatic cells with green fluorescent protein (GFP) enabled the authors to conclude that regenerating β‐cells have multiple cellular origins. Nascent β‐cells emerged from the neurod‐positive pancreatic endocrine cells after a near‐total ablation, and divided into subpopulations according to their different cellular origins. Moreover, intrapancreatic ductal cells resisted giving rise to regenerating β‐cells (Wang, Li et al., 2020, Wang, Du et al., 2020), whereas transdifferentiation of α‐cell and δ‐cell can regenerate β‐cells (Wang, Wei et al., 2020, Wang, Yang et al., 2020).

#### 2.3.2 Pancreatectomy

The inactivation of β‐cells to induce T1DM can also be achieved through the surgical ablation of the pancreas (pancreatectomy). Although it is technically difficult to perform, the expression of GFP under the control of the insulin promoter facilitates the physical removal of the pancreas (Table 3). Delaspre's study compared β‐cell ablation through pancreatectomy using chemical and genetic approaches that were previously reported using STZ injection and NTR/MTZ ablation system (Delaspre et al., 2015). The ability of zebrafish to regenerate a functional pancreas after surgical ablation presented different patterns of cell proliferation in islets and ducts according to the method used. Partial physical ablation was achieved by pancreatectomy (Delaspre et al., 2015), which resulted in an increased division of existing β‐cells that was not observed after chemical ablation, suggesting that as surgical removal affects many more cell types than β‐cells, it may have triggered a distinct set of signals that in aggregate lead to β‐cell proliferation. These kinds of adult zebrafish models of diabetes set a precedent for defining the roles of dividing cells as well as pancreatic ducts during β‐cell regeneration.

#### 2.3.3 Dietary induction

The strategy to induce transgenic lines with fluctuating, constant or stepwise increase of HG concentrations, HFD, or a combination of HFD with HG has allowed the development of interesting models (Table 3). In this vein, various genetic zebrafish models have been used to study the effect on the newborn of diabetic conditions induced by a HG diet, such as retinopathy (Alvarez et al., 2010), bone imbalanced metabolism (Carnovali et al., 2016), cerebrovascular patterning, neurovascular coupling (Chhabria et al., 2020), heart failure (Kim et al., 2023) and even high glucose during gestational hyperglycaemia. Among retinopathy models, a pioneer study characterized a novel model of non‐proliferative diabetic retinopathy in adult zebrafish, in which the transgenic line *Tg(fli1:EGFP)y* was subjected to oscillating hyperglycaemia (Alvarez et al., 2010). As a result of oscillating glucose, the retinas of hyperglycaemic zebrafish displayed many of the pathophysiological aspects of human diabetic retinopathy (DR). Thus, the authors proposed that debilitating blindness associated with DR may be halted by neuroprotection of cones. After this study, another study reported a novel model of DR that was attained by immersing the transgenic *flk:EGFP* line in 130 mM glucose for 3 days (Xu & Wu, 2021). The model allowed study of diabetic retinal vascular dysfunction; zebrafish larvae presented dilatation of hyaloid‐retinal vessels that was accompanied by morphological lesions with disruption of tight junction proteins, overproduction of vascular endothelial growth factor (VEGF) mRNA and increased NO production. It offered the advantage of analysing the phenotype in 3 days, compared to the 30 days proposed by the model of Alvarez et al. (2010). A more recent study approached a diabetic retinopathy model by exposing zebrafish embryos of the transgenic *Tg(flk:EGFP)* line at high constant glucose concentration (Alvarez et al., 2010). Embryotoxicity and morphological changes associated with hyperglycaemia were dose dependent, with the highest glucose concentration evaluated (160 mM) as the most harmful, inducing the largest number of morphological changes and the highest expression of diabetic retinopathy inflammatory markers.

Until Carnovali et al. (2016) created a new T2DM adult animal model in which to study the resulting bone disease in zebrafish, there was not an animal model able to recapitulate all the characteristics of diabetes‐induced skeletal affection and fragility seen in humans (Carnovali et al., 2016). It is well known that chronic hyperglycaemia affects bone health causing fragility, mechanical strength reduction and increased propensity to fracture because of impaired bone matrix microstructure and aberrant bone cell function (Wen et al., 2021). T2DM was induced in adult male zebrafish by glucose administration in the water (Carnovali et al., 2016), which showed an imbalance in bone metabolism; fish were unable to depose new mineralized matrix and showed bone resorption lacunae associated with an intense osteoclast activity as well as alterations in bone‐specific markers such as decreased alkaline phosphatase activity and increased tartrate‐resistant acid phosphatase activity. On the other hand, to examine the chronic effect of glucose exposure on cerebrovascular patterning and neurovascular coupling, a novel non‐invasive, non‐anaesthetized larval zebrafish model was recently developed (Chhabria et al., 2020). Zebrafish expressed three transgenes, *kdrl:mCherry*, *gata1:DsRed* and *nbt:GCaMP3*, which allowed simultaneous quantification of neuronal activation, cerebrovascular anatomy and red blood cell speed in individual cerebral vessels. On the other hand, the exposure to high glucose of the zebrafish expressing the *claudin5a:GFP* reporter allowed us to determine that the effects of glucose were mediated via alteration of the BBB (Chhabria et al., 2020). The claudin5a protein fused to GFP was significantly reduced by glucose exposure in the tectal vessels, suggesting that the negative effects of glucose on cerebrovascular development and neurovascular coupling may be mediated, at least in part, by alteration of BBB structure or integrity. This is the first non‐mammalian model of neurovascular coupling that offers a potential strategy to ameliorate the effects of hyperglycaemia on cerebrovascular function.

A zebrafish larvae model was established for in vivo studies of diabetes mellitus with heart failure with reduced ejection fraction (DM‐HFrEF) by sequentially treating them with a combination of d‐glucose (Kim et al., 2023), STZ and terfenadine, a potassium channel blocker. Diabetic zebrafish treated with terfenadine showed significantly less myocardial fractional shortening, more irregular contractions, significant increase in the levels of natriuretic peptide B (a heart failure biomarker), markedly reduced motility and reduced survival rates (Larijani et al., 2021; Kim et al., 2023). The importance of this novel model relies in the possibility of performing mechanistic studies to understand the pathophysiology of DM‐HFrEF (Kim et al., 2023).

On the other hand, Yin et al. (2015) developed a feasible, rapid and straightforward technique for determining the mechanism of gene function using conditional mutagenesis in zebrafish induced with a high‐fat diet. The transgenic expression of both Cas9 and multiple sgRNAs led to specific gene inactivation. In the study, they achieved the liver‐specific knockdown of the insulin receptors a and b (*insra* and *insrb*) and tyrosinase inactivation, with the corresponding hyperglycaemic phenotype and defects in the retinal pigment epithelium. Some studies proposed a model of diabetic vascular complications similar to those of early atherosclerotic vascular injuries in mammals by exposing transgenic zebrafish lines to a HFD + HG diet for 10 days (details in Table 3) (Wang et al., 2013; Wongdee & Charoenphandhu, 2015). The diabetic zebrafish larvae significantly increased tissue levels of insulin, glucagon, glucose, total triglyceride and cholesterol, developed thickened endothelial layers, distinct perivascular lipid depositions, substantial accumulations of inflammatory cells in the injured vasculature, and a decreased velocity of blood flow. Overfeeding the Tg(−1.0ins:EGFP)^sc1*^ zebrafish line expressing the enhanced GFP (EGFP) in a dominant‐negative IGF‐I receptor in skeletal muscle led to the well‐known diet‐induced obesity model. This zebrafish presented a hyperglycaemic phenotype, and allowed for demonstrating that T2DM zebrafish shared pathological pathways with humans (Wang et al., 2013).

#### 2.3.4 *pdx1* mutants

The *pdx1* homozygous mutant zebrafish was a novel and clinically relevant model for diabetic retinopathy at the date it was reported (Table 3) (Kimmel et al., 2015). The *pdx1* gene is a pancreatic transcription factor that in humans is linked to genetic forms of diabetes, thus allowing the elucidation of the mechanisms behind hyperglycaemic pathologies. In contrast to previous models limited to short‐duration treatments and assessing phenotypes at a single time point, this mutant constituted a novel DR model that manifested a longer duration diabetic state, as well as key diabetic features such as reduced β‐cells, decreased insulin and elevated glucose from larval through adult stages. All these features make the *pdx1* mutant uniquely suitable among zebrafish models for studying the long‐term effects of hyperglycaemia.

In a recent study, vessel morphology was examined in a *pdx1* mutant expressing the *fli1a:EGFP* transgene (Ali et al., 2020). This mutant exhibited clear vascular phenotypes and disease progression, including arterial vasculopenia, capillary tortuosity and hypersprouting, which could be detected over more than 1 year. *pdx1* mutant zebrafish constitute a valuable complement to rodent and other mammalian models of DR, in particular for research into the mechanistic interplay of diabetes with vascular and neuroretinal disease. Moreover, recent studies reported on the diabetic *pdx1* ^−/−^ zebrafish mutant as a novel model to study mechanisms of hyperglycaemia‐induced retinopathy and diabetic nephropathy (DN) (Kimmel et al., 2015; Quiroz & Yazdanyar, 2021). For the DR model, the mutant was obtained through CRISPR/Cas9‐mediated knockout of the transcription factor *pdx* in specific transgenic lines (mentioned in Table 3). As a result, larval mutants showed vasodilatation of blood vessels through increased vascular thickness in the hyaloid network, whereas adult mutants impaired in Glu homeostasis had increased hyperbranching and hypersprouting with new vessel formation in the retina. On the other hand, for the DN model, the *pdx1* mutation was induced in transgenic lines (mentioned in Table 3) that allowed identifying signs of early disease progression in the larval stage such as glomerular hypertrophy, impairments in the filtration barrier corresponding to microalbuminuria and glomerular basement membrane thickening, and several selective features of later diabetic kidney damage in adult mutants, such as progressive glomerular basement membrane thickening and glomerular hypertrophy (Kimmel et al., 2015; Wang, Wei et al., 2020, Wang, Li et al., 2020, Wang, Du et al., 2020, Wang, Yang et al., 2020; Xu & Wu, 2021).

#### 2.3.5 Hypoxia induction

Pathological angiogenesis in the retina is the leading cause of human blindness resulting from DR, age‐related macular degeneration and retinopathy of prematurity (Table 3) (Cao et al., 2010). A hypoxia‐induced model using the fluorescent zebrafish *Tg(fli1:EGFP)y* was developed to reproduce retinal angiogenesis that is normally induced during diabetic retinopathy. Adult *fli1:EGFP* zebrafish were placed in hypoxic water for 3−10 days, and hypoxia further induced neovascularization in a predictable area of capillary plexuses that were identifiable with GFP imaging (Cao et al., 2008).

## 3 DRUG DISCOVERY IN ZEBRAFISH DIABETIC MODELS

The zebrafish model is a bridge between in vitro assays and mammalian in vivo studies because it allows the drug discovery industry to minimize unpredicted human safety events in clinical trials with diligent preclinical safety testing at a relatively low cost in a vertebrate whole‐animal model (Cao et al., 2010; Cassar et al., 2019; Patton et al., 2021). The conserved organs, physiology and morphology of zebrafish, specifically the brain, kidney, gastrointestinal tract and circulatory system, are utilized to visualize and analyse the therapeutic impact of drugs on their growth and development for assessing human risk and for preclinical drug discovery and screening. Particularly for diabetic drug discovery, numerous zebrafish models have been developed and validated. Hereafter, we mention those models that have been proposed as platforms for drug screening and those further validated using chemical U.S. Food and Drug Administration (FDA)‐approved anti‐diabetic drugs, such as metformin, glipizide, rosiglitazone, tolbutamide, pioglitazone, glimepiride, sunitinib, PK11195, meclizineo, trigonelline and the nitric oxide donor sodium nitroprusside (Table 4) (Navik et al., 2022; Zhang et al., 2003).

**TABLE 4:** Zebrafish diabetic and hyperglycemic pathologies models for drug discovery.

Pathology model	Method	Drug	Outcome	Mechanism	Reference
Diabetes	Glucose exogenous exposure + cAMP+DEX treatment	Metformin, glipizide, and rosiglitazone	Reduction of the high blood glucose levels in larvae	Co‐treatment with cAMP+DEX resulted in the significant decrease of PEPCK expression	Ayobahan et al., 2020
	Immersion in chronic Glu	Glimepiride and metformin	Reversion of hyperglycaemia	Probable increase of peripheral tissues response to insulin and not by pancreatic secretion of insulin, since exogenous insulin was ineffective	Navik et al., 2022
	*Pdx1* mutant	Tolbutamide	Amelioration of hyperglycaemia in embryos, larvae and juvenile zebrafish	The drug induced the direct decrease of Glu levels in *pdx1* mutants probably through insulin‐stimulated glucose utilization	Zhang et al., 2003
	Insulin injection	Pioglitazone	Reversion of hyperglycaemia	Recovery of pancreatic islets and islet β‐cells, probably by targeting the calcium and MAPK signalling pathways	Ayobahan et al., 2020
Diabetes and DIO	InsGFP zebrafish + overfeeding	Glimepiride and metformin	Amelioration of hyperglycaemia in adult zebrafish	New insights into the mechanism not mentioned	Patton et al., 2021
Diabetic vasculopathy	Immersion in HFD + HG	Pioglitazone and metformin	Attenuation of early vascular changes in larvae	Partial inhibition of the endothelial thickening, inflammatory activation and lipid accumulation in the caudal vein. However, drugs did not restore the decreased blood flow caused by the HCD‐HG	Cao et al., 2010
Diabetes‐related affective pathogenesis	Immersion in HFD + HG	Not tested	Linked the affective CNS pathogenesis in zebrafish to DM‐evoked pathophysiological phenotypes	T2DM metabolic symptoms were accompanied by robust anxiety‐like behaviour, elevated cortisol, and altered central and peripheral cytokines, neurotrophin‐related and apoptotic genes	Larijani et al., 2021
Diabetic hearing loss	Immersion in Alloxan	Trigonelline	Enhanced sensory function of altered auditory electrophysiological responses in larvae zebrafish	Trigonelline binds to the active site of NGF, and significantly reduces hair cell loss and neuromast damage, and increases the number of neuromasts in diabetic zebrafish	Ayobahan et al., 2020
Diabetic‐heart failure	Immersion in Glu + STZ	DM zebrafish were treated with TER to induce HF	Severe cardiac dysfunction	Significantly less myocardial fractional shortening and more irregular contractions	Larijani et al., 2021
Diabetic retinopathy model	Immersion in HG of the transgenic *flk:eGFP* zebrafish line	Not tested	A potential platform to test drugs for disorders of retinal vessels	Dilatation of hyaloid‐retinal vessels accompanied by morphological lesions	Metzner et al., 2020
Diabetic retinopathy and bone imbalanced metabolism model	Immersion in HG of the transgenic *kdrl:GFP* zebrafish line	Not tested	A potential platform to elucidate in vivo the molecular mechanisms of metabolic changes, which influence the bone tissues regulation in human diabetic patients	Abnormal retinal blood vessels, resembling human diabetic retinopathy/bone loss and altered bone cell function	Pringle et al., 2019

Abbreviations: CNS, central nervous system; DM, diabetes mellitus; Glu, glucose; HBG, high blood glucose; HCD, high cholesterol diet; HG, high glucose; NGF, nerve growth factor; TER, terfenadine.

## 4 DRUGS FOR DIABETES

Many of the models described in this review have also been validated for drug discovery and testing for diabetes and diabetic complications. One study demonstrated that metformin, glipizide and rosiglitazone reduced the high blood glucose levels in zebrafish larvae by reducing significantly the expression of PEPCK (Navik et al., 2022). Given that chronic hyperglycaemia activates PEPCK expression and ultimately gluconeogenesis, one critical function of potential antidiabetic drugs is to reduce PEPCK expression (Table 4). Similarly, treatment with glimepiride and metformin could reverse the hyperglycaemic state of adult zebrafish (Patton et al., 2021). Treatment with the sulfonylurea anti‐diabetic drug tolbutamide could reduce the high glucose level in *pdx1* mutant embryos, larvae and juvenile zebrafish, using a promising model that allows evaluation of pharmacological effects and toxicities of new antidiabetic drugs (Zhang et al., 2003). In a larval insulin‐resistance model, the pancreatic islets and β‐cells were recovered after treatment with the insulin‐resistance drug pioglitazone, and insulin‐resistance drugs had as significant an effect in zebrafish as in humans (Ayobahan et al., 2020). Altogether, these results proved the value of this zebrafish insulin‐resistance model for drug testing and drug discovery in insulin resistance and diabetes. In addition, in the diabetic diet‐induced obesity model, Zang et al. (2017) developed a platform for mechanistic and therapeutic studies of diet‐induced glucose intolerance, insulin resistance and drug discovery for the treatment of human obesity and DM (Cao et al., 2010). The protocol, previously validated with some modifications in regards to the alloxan doses administered to larvae, tested the effect of natural extracts derived from *Platycodon grandiflorus* aerial parts compared to the effect of the FDA‐approved drugs glimepiride. Effectively, glimepiride increased both pancreatic islet size and glucose uptake in alloxan‐treated zebrafish, thus validating the model for further drug screening. A new and useful tool for further research on drug discovery and toxicity research against diabetes was developed by Mohammadi et al. (2020). The T2DM zebrafish model also exhibited insulin resistance, and treatment with metformin for 20 days produced a significant improvement in blood glucose and inflammation caused by high concentrations of glucose. This new combination therapy for T2DM was effective and fast and was proposed to be used for the early phase of T2DM research (Cao et al., 2010; Cassar et al., 2019; Navik et al., 2022).

## 5 DRUGS FOR DIABETIC PATHOLOGIES

In the model of diabetic vascular complications developed by Wang et al. (2013), the early vascular changes were attenuated when pioglitazone or metformin was administered at the same time as the HCD‐HG treatment (Table 4). Both drugs partly inhibited endothelial thickening, inflammatory activation and lipid accumulation in the caudal vein, but did not restore the decreased blood flow caused by the HCD‐HG. The promising T2DM larval zebrafish model developed by Jung et al. (2016) allowed for screening and drug discovery for diabetic retinopathy and particularly for disorders of retinal vessels related to disruption of tight junction proteins and excessive VEGF and nitric oxide production. The model provided two advantages over retinopathy models reported previously, including short incubation time (3 days) and low incubation volume, which means that less drug is needed per test. This model is much more time‐efficient than previous models that reported longer incubation periods of 11 and 30 days. Likewise, the hypoxia‐induced retinopathy fli‐EGFP zebrafish model allowed us to assess the therapeutic efficacy of orally active antiangiogenic drugs (Navik et al., 2022; Zhang et al., 2003). The anti‐VEGF compound sunitinib or ZN323881 could significantly inhibit retinal neovascularization, suggesting that VEGF is responsible for hypoxia‐induced angiogenesis in adult zebrafish. The embryonic model of the transgenic *Tg(flk:EGFP)* zebrafish reported by Lee and Yang (2021) can be used to evaluate the early stages of toxicity and efficacy of drugs quickly, facilitating the screening of new drug candidates for treatment of diabetic retinopathy. For instance, aflibercept 100 μg/mL efficacy was confirmed, as it could improve the hyperglycaemia‐increased expression of diabetic retinopathy inflammatory markers and the diameters of retinal vessels in the zebrafish eye. Moreover, the *pdx1* mutant is a valuable resource for molecular studies to identify new targets for the treatment of early as well as late diabetic retinopathy (Larijani et al., 2021). In the *pdx1* ^−/−^ zebrafish mutant DR model, both vascular aspects responded to antiangiogenic and antihyperglycaemic pharmacological interventions in the larval stage, and were accompanied by alterations in nitric oxide metabolism. The antihyperglycaemic drugs metformin (10 μmol/l) and PK11195 (10 μmol/l) lowered glucose concentration in larvae, with PK11195 showing a stronger effect on glucose levels compared with metformin. The antiangiogenic drug l‐*N* ^G^‐nitro‐l‐arginine methyl ester (l‐NAME, 10 μmol/l) significantly rescued the proangiogenic aspects of hyperbranching and hypersprouting as well as the increased vascular diameters in the *pdx1* ^−/−^ hyaloid network. In the *pdx1* ^−/−^ zebrafish mutant diabetic nephropathy model, pharmacological intervention with *N*‐(*p*‐amyl cinnamoyl)anthranilic acid and meclizine revealed the contribution of phosphatidylethanolamine in the early establishment of kidney damage (Kimmel et al., 2015). Treatment of diabetic zebrafish larvae with trigonelline, on the other hand, resulted in improved sensory function in a prospective pharmacological model for treating diabetic hearing loss (Kimmel et al., 2015). The authors demonstrated that nerve growth factor was involved in the mechanism of action of trigonelline in recovering auditory function, providing compelling evidence of this FDA‐approved drug's protective effects on auditory damage while also indicating the value of this approach as an effective therapeutic alternative for treating diabetic hearing loss.

Additionally, a potential platform for modelling other diabetic metabolic disorders, their comorbid central nervous system conditions, and for searching novel anti‐diabetic, anti‐stress or ‘combined’ (anti‐diabetic/anti‐stress) drugs was proposed by Wang, Wei et al. (2020, Wang, Li et al., 2020, Wang, Du et al., 2020, Wang, Yang et al., 2020), but not further validated with any drug treatment (Wang et al., 2013). In the *claudin5a:GFP* reporter transgenic zebrafish, the reduced expression of GFP in the tectal vessels due to high‐glucose exposure was rescued by co‐treatment with the nitric oxide donor sodium nitroprusside. Administration of sodium nitroprusside resulted in a marked reversal of vascular patterning and neurovascular coupling defects, suggesting that diabetes‐associated neurovascular and cerebrovascular defects may be treatable by nitric oxide donating drugs.

Finally, there is an urgent need for new, simple, rapid and cost‐effective methods to evaluate the effects of hypoglycaemic agents on the cardiovascular system, particularly during the early developmental stages. In this vein, a recent and easy‐to‐use zebrafish model of dilated cardiomyopathy induced by a brief treatment with terfenadine allows for assessing in vivo the effects of new medications for DM and heart failure. Its feasibility was proposed in the screening of new drugs for diabetic HF.

## 6 FUTURE PERSPECTIVES

In the realm of endocrine disease research using zebrafish models, future investigations should focus on elucidating specific molecular pathways that mirror human conditions. This deep dive could yield vital insights into disease mechanisms and unveil novel therapeutic targets. Additionally, the integration of state‐of‐the‐art technologies such as CRISPR/Cas9 for genome editing (Ayobahan et al., 2020; Larijani et al., 2021) and high‐throughput screening in zebrafish research could revolutionize our understanding of disease progression and the efficacy of treatments, potentially leading to ground‐breaking therapeutic strategies for diabetes and other hyperglycaemic diseases (Metzner et al., 2020; Wu & Wang, 2020). Comparative studies between zebrafish and other model organisms, like rodents, are also crucial. These studies could reveal unique and shared pathways in disease mechanisms, thereby enhancing the translational value of research findings (Astone et al., 2017; Pringle et al., 2019). Moreover, conducting longitudinal studies using zebrafish models will be instrumental in understanding the stages of endocrine disease progression, which is critical for identifying effective intervention points and comprehending the long‐term effects of potential treatments.

## 7 CONCLUSION

This review underscores the urgent need for a multidisciplinary approach that blends genetics, proteomics and pharmacology for developing innovative anti‐diabetic drugs. Such collaborative research efforts are essential for breakthroughs in understanding and treating diabetes and its complications. The promising outcomes from zebrafish model studies highlight the potential of these models in the realm of personalized medicine. Future research should pivot towards developing patient‐specific zebrafish models to test the efficacy and safety of drugs, thereby customizing treatments to individual genetic profiles. Lastly, there is a pressing need for increased funding and resources dedicated to diabetes research using zebrafish models. With adequate support, these models could significantly hasten the pace of discovery in understanding and treating diabetes and hyperglycaemic diseases, leading to faster and more effective therapeutic solutions.

## AUTHOR CONTRIBUTIONS

Tamsheel Fatima Roohi: Conceptualization (lead); writing original draft (lead); formal analysis (lead); writing, review and editing (equal). Syed Faizan: writing original draft (lead); review and editing (equal). Mohd. Farooq Shaikh: Conceptualization (supporting); review and editing (equal). Kamsagara Linganna Krishna: Conceptualization (supporting); review and editing (equal). Seema Mehdi: Conceptualization (supporting); review and editing (equal). Nabeel Kinattingal: Conceptualization (supporting); review and editing (equal). Alina Arulsamy: Conceptualization (supporting); review and editing (equal). All authors have read and approved the final version of this manuscript and agree to be accountable for all aspects of the work in ensuring that questions related to the accuracy or integrity of any part of the work are appropriately investigated and resolved. All persons designated as authors qualify for authorship, and all those who qualify for authorship are listed.

## CONFLICT OF INTEREST

The authors declare that they have no known competing financial interests or personal relationships that could have appeared to influence the work reported in this paper.

## FUNDING INFORMATION

The authors declare that no funds, grants, or other support were received during the preparation of this manuscript.
